# Crystal facet engineering in perovskite photovoltaics: the rise of (111)-oriented structures

**DOI:** 10.3389/fchem.2025.1692350

**Published:** 2025-12-01

**Authors:** Feng Gao, Pu Zhao, Xiaojun Qin, Zhiguo Zhao

**Affiliations:** Photovoltaic Technology Department, Huaneng Clean Energy Research Institute, Beijing, China

**Keywords:** perovskite solar cell (PSC), facet engineering, stability, power conversion efficiency (PCE), photovoltaics

## Abstract

Perovskite solar cells (PSCs) are regarded as a promising candidate for next-generation photovoltaics. Facet engineering for the controlled growth of perovskite crystals has emerged as a breakthrough strategy to address the efficiency-stability trade-off in PSC devices. Among various crystallographic orientations, (111)-oriented perovskite films have garnered significant attention due to their unique advantages in defect tolerance, ion migration suppression, and environmental adaptability. This review systematically summarizes the structural, electronic, and stability characteristics of the (111) facet. By analyzing key engineering strategies such as additive-regulated growth, ligand-assisted crystallization, and substrate-template induction, the roles of these methods in suppressing competitive facet orientations and promoting preferential (111) alignment are revealed. Experimental evidence demonstrates that (111)-dominated orientations exhibit superior stability in perovskite films across different bandgaps, making them ideal for both single-junction perovskites and tandem devices. Despite notable progress, challenges remain in scaling up (111)-predominant films with homogeneous morphology and reconciling growth kinetics with thermodynamic stability. Emerging solutions, such as machine learning-guided additive design and *in situ* characterization of facet-dependent degradation, are highlighted as critical pathways to unlock commercial viability. By bridging fundamental crystallography and device performance, this review provides a roadmap for leveraging (111) facet engineering to unleash the full potential of PSCs.

## 1 Introduction

Since their debut in 2009, perovskite solar cells (PSCs) have risen to prominence as one of the most promising next-generation photovoltaic technologies, boasting a remarkable power conversion efficiency (PCE) leap from 3.8% to over 27% (Gao et al., 2020; Green et al., 2025; Li J. et al., 2025; Li Y. et al., 2025; Luo et al., 2022). This progress is fueled by their exceptional light absorption coefficients, high charge carrier mobility, and cost-effective solution processability. This leap forward primarily stems from obtaining dense, pinhole-free films, continuous refinement of defect passivation strategies, and iterative optimization of device architectures (Cai et al., 2025; Chen et al., 2025; Tian and Zhang, 2025; Zhang P. et al., 2025; Zou et al., 2024). However, insufficient long-term operational stability and the efficiency loss in large-area devices remain the core bottlenecks hindering commercialization (Feng et al., 2025; Mazumdar et al., 2021; Qu et al., 2024; Yan et al., 2025; Zhan et al., 2025). The research community increasingly recognizes that traditional “composition-defect” modulation is nearing its limits, urging the exploration of new performance-enhancing paradigms from a crystallographic perspective.

Facet engineering has attracted significant attention as an effective strategy for precisely tailoring the microstructure of perovskite films (Fang et al., 2022). Owing to differences in atomic arrangement, surface energy, and electronic structure, various crystal facets of perovskite—such as (001), (011), and (111)—exhibit anisotropic physical and chemical properties (Fu et al., 2025; Zhang et al., 2023; Zhu et al., 2023). This anisotropy exerts a decisive influence on carrier dynamics, trap state density, and ultimately the overall performance and operational lifetime of the devices (Gao and Zhao, 2023). The goal of facet engineering is to controllingly direct the growth orientation of perovskite crystals, thereby preferentially exposing specific facets with superior optoelectronic properties and chemical stability. This approach optimizes film quality, reduces defects, improves charge transport, and enhances the overall device performance. As such, precise control over crystal facet orientation is regarded as the next pivotal lever—following advances in defect passivation—for driving improvements in both the efficiency and stability of PSCs.

Among various low-index facets, the (111) orientation has attracted considerable interest due to its unique potential for reconciling high stability with efficiency (Ma et al., 2023b; Zhang et al., 2025). Early studies revealed that the Pb-I bond arrangement on the (111) facet is denser, and its surface energy is lower than that of the (001) facet, granting it enhanced resistance to moisture adsorption and thermal phase transitions (Ma et al., 2023a; Zhang Y. et al., 2025). Moreover, its exposed halogen atoms can reduce surface defect density, thereby suppressing non-radiative recombination. However, the growth of the (111) facet is constrained by thermodynamic and kinetic equilibria, making its directional growth challenging. Often, achieved films contain a high proportion of randomly oriented facets alongside the (111) orientation, resulting in insufficient facet purity (Zhou et al., 2025). Recent advances in additive design, intermediate-phase regulation, and anti-solvent engineering, have preliminarily achieved preferential (111) growth, validating its significant positive impact on device performance.

Despite these promising developments, (111) facet engineering still confronts several challenges, including an unclear competitive growth mechanism, difficulty in controlling facet distribution in large-sized films, and a lack of systematic structure-property correlation. To address this knowledge gap, this review comprehensively summarizes the research progress on the (111) crystal facet orientation in PSCs. We begin by elucidating the crystallographic and optoelectronic properties of the (111) facet, followed by a detailed analysis of strategies for its controlled growth across different fabrication methodologies. We then discuss the underlying mechanisms through which this facet influences device efficiency and stability. Finally, we outline prevailing challenges and future research directions. This review aims to clarify current advancements and bottlenecks, providing a valuable reference for designing future facet engineering protocols and mapping their industrialization path.

## 2 Crystallographic and optoelectronic properties of the (111) facet

**FIGURE 1:**
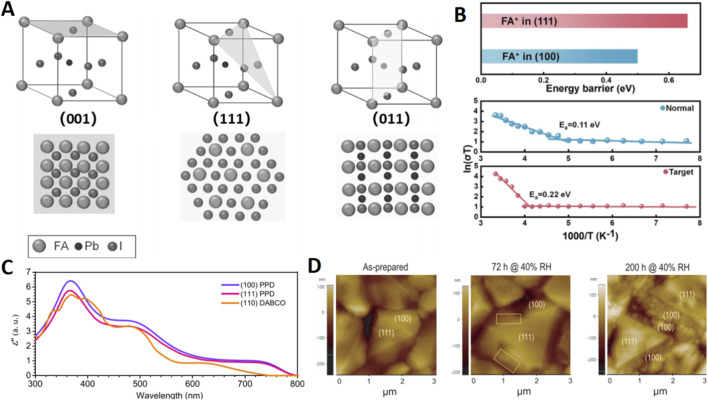
**(A)** Atomic arrangements of perovskite crystal facets on an ideal perovskite cubic crystal: (100), (111), and (110) facets. **(B)** Active migration energy of cationic migration in the (100) and (111) facets, temperature-dependent conductivity measurements of normal and target perovskite films under ambient conditions with 35% RH and 25 °C. **(C)** Calculated imaginary part of dielectric constant in the range of UV-vis-NIR spectrum of a-FAPbI_3_ with different facets. **(D)** AFM images of the as-prepared sample.

The performance of PSCs is profoundly influenced by the surface properties of their constituent crystals, which vary significantly with orientation. Common facets in perovskites, including (001), (011), and (111), exhibit distinct physicochemical behaviors due to differences in atomic arrangement and termination. The (001) facet is typically terminated by Pb-I or FA-I layers, possessing high symmetry and atomic density that render it one of the most thermodynamically stable low-index surfaces (Ma et al., 2022a). In contrast, the (011) facet exhibits a combination of half organic cations and half halogen ions, resulting in reasonable surface charge neutrality but unsaturated atomic coordination. The (111) facet, terminated by 3/8 organic cations and 3/2 halogen ions, possesses a negatively surface charge and even less saturated coordination, leading to a relatively higher surface energy (Figure 1A). These inherent differences in surface structure fundamentally direct crystal growth kinetics and ultimately determine the orientation distribution within the polycrystalline film.

From a crystallographic standpoint, the (111) facet represents a high-Miller-index plane in the perovskite cubic phase. Its larger lattice spacing (d-spacing ≈7.3 Å compared to ≈6.4 Å for the (001) facet) indicates a more open surface structure. This openness creates a more tortuous pathway for ion migration, which is quantified by a significantly higher activation energy for FA^+^ ion migration on the (111) facet (1.51 eV) than on the (001) facet (1.13 eV). This inherent property effectively suppresses detrimental ion migration and phase segregation under operational stressors like illumination or electric fields (Figure 1B).

The optoelectronic characteristics of the (111) facet are equally remarkable. Calculations based on equilibrium electron density indicate a higher dielectric constant for the (111) facet, which aids in reducing exciton binding energy and promoting efficient charge carrier separation (Figure 1C) (Ma et al., 2022b). Further reinforcing this advantage, the ordered stacking inherent to (111)-oriented films results in fewer grain boundaries and a lower trap state density (Li et al., 2025). This superior microstructure translates directly into enhanced photophysical properties, yielding extended carrier lifetimes up to 283 ns and longer diffusion lengths, substantially exceeding those of randomly oriented films.

Perhaps most critically, the (111) facet exhibits exceptional stability—a paramount concern for PSCs. Ma et al. provided compelling visual evidence through *in situ* AFM, showing that under humidity exposure (30%–40% RH), the (001) facet developed δ-phase protrusions within 72 h and severe fragmentation after 200 h, while the (111) facet remained virtually unchanged (Figure 1D) (Ma et al., 2023b). Quantitative analysis revealed that the adhesion energy for water molecule adsorption on the (111) facet (85.12 mN/m) is lower than on the (001) facet (93.08 mN/m), and its Pb-I bond structure is inherently more robust. These findings have been consistently corroborated by subsequent work from multiple groups, solidifying the consensus that the (111) facet, through its innate ability to suppress ion migration, reduce surface defects, and enhance chemical resilience, represents a cornerstone strategy for achieving long-term stable PSCs.

In addition to the enhanced moisture stability previously discussed, the (111) facet also demonstrates exceptional resilience against UV light irradiation, and operational stresses. *In situ* optical microscopy, electroluminescence (EL) imaging, photovoltage (PLV) hysteresis, and open-circuit voltage decay (OCVD) measurements collectively reveal that the activation energy for FA^+^ migration on the (111) facet is twice that of the (100) facet—indicating intrinsically suppressed ion migration (Qu et al., 2025). Under UV light soaking for 1,500 h, the (001) facet undergoes significant degradation with marked PbI_2_ formation, whereas the (111) facet remains virtually unchanged. Furthermore, devices dominated by the (111) facet retain 91% of their initial efficiency after 720 h at 85 °C and demonstrate 95% retention after 3,500 h of maximum power point (MPP) tracking, far exceeding the stability of (100)-oriented devices. These findings confirm that the (111) facet offers comprehensively superior stability under multiple stressors including humidity, heat, light, and electrical bias.

## 3 Strategies for achieving high-purity (111) facet orientation

The pursuit of high-purity (111) facet orientation is driven by its compelling properties: dense atomic packing, high dielectric constant, and a high energy barrier against ion migration. These characteristics position it as a key enabler for high-efficiency, stable PSCs. The various strategies for achieving (111)-oriented perovskite films and their corresponding device performance are comprehensively summarized in Table 1 for a clear comparison. The following sections detail the primary strategies for achieving this coveted orientation, organized by fabrication methodology and device structure, and elucidate their underlying mechanisms.

**TABLE 1:** Summary of regulating methods and photovoltaic performance of (111) oriented perovskite solar cells.

Regulating material/Method	Device architecture	Performance metrics				Perovskite preparation	Device stability	E.g., (eV)	Mechanism	Refs
		V_OC_(V)	J_SC_ (mA/cm^2^)	FF(%)	PCE(%)					
Diethylamine hydrochloride (DEACl) and formamide (Fo) (in PVK)	FTO/bl-SnO_x_/SnO_2_/perovskite/CH_3_COOK/spiro-OMeTAD/Au	1.14/1.15	25.88/26.40	81.45/83.05	24.03/25.21	Two-step	MPP T91 @ 1080 h	1.49	Thermodynamic + Dynamic	Adv. Mater. 2025, 37(20), 2418008
Melamine(MEA) and CsI (in PbI_2_)	ITO/NiO_x_/Me-4PACz/(FACsPbI_3_)_x_(MAPbI_3_)_y_/PEAI/PCBM/BCP/Ag	1.158/1.199	24.64/25.56	77.2/83.7	22.03/25.66	Two-step	MPP T90 @ 1000 h	1.54	Thermodynamic	Energy Environ. Sci. 2025, 18(5), 2,436–2,451
N-butylammonium iodide (BAI)、n-butylammonium acetate (BAAc) (in PbI_2_)	ITO/SnO_2_/perovskite/FPEAI/spiro-OMeTAD/Au	1.144/1.189	24.95/25.65	77.69/82.73	22.18/25.23	Two-step	MPP T93 @ 1,500 h	∼1.54	Dynamic	Energy Environ. Sci. 2024, 17(16), 6058–6067
Benzimidazolium Chloride (BNCl) (in PbI2)	FTO/SnO_2_/perovskite/spiro-OMeTAD/Au	1.150/1.159	25.59/26.05	82.74/83.65	24.34/25.27	Two-step	MPP T80 @ 2,500 h	∼1.54	Thermodynamic	Zhou et al. (2025)
Tea saponin (TS) (in SnO_2_)	ITO/SnO_2_/perovskite/PEAI/spiro-OMeTAD/MoO_3_/Ag	1.159/1.172	24.8/24.9	80.4/82.8	23.1/24.2	Two-step	20% RH T90@ 800 h	1.51	Interface induction	Ouyang et al. (2024)
Piperidine (PPD) (in PVK)	FTO/SnO_2_/perovskite/spiro-MeOTAD/Au	1.15/1.17	24.84/25.38	81.02/82.98	23.14/24.64	One-step	Lignt- soaking T96 @ 1000 h	1.5	Dynamic	Joule 2023, 6, 2,626–2,643
Cyclohexylamine (CHA) (in PVK)	FTO/SnO_2_/perovskite/spiro-MeOTAD/Au	1.12/1.15	25.6/25.3	84/82	24.0/23.8	One-step	30%–40% RH T95 @ 2000h	1.5	Thermodynamic	Science 2023, 379(6628), 173–178
Isobutanol (IBA) (as antisolvent)	ITO/TiO_2_/perovskite/Spiro-OMeTAD/Au	1.02/1.06	25.05/25.09	79.49/82.95	20.31/22.03	One-step	Storage T90@ 2000 h	∼1.56	Thermodynamic + Dynamic	Solar Rrl 2022, 6(4), 2100973
IBA/isopropanol (IPA) (as antisolvent)	ITO/SnO_2_/PCBM/perovskite/Spiro-OMeTAD/Au	1.14/1.16	23.67/24.49	76.05/77.85	20.44/22.09	One-step	MPP T100 @ 600 h	1.55	Interface induction + Dynamic	Adv. Mater. 2023, 35(28), 2301115
Water (H_2_O) (in antisolvent)	FTO/TiO_2_/perovskite/MeO-PEAI/spiro-MeOTAD/Au	1.174/1.187	26.11/26.21	83.39/83.44	25.6/26.0	One-step	MPP T95 @ 3,500 h	∼1.55	Interface induction + Dynamic	Angew. Chem. 2025, 137(4), e202415949
	FTO/Me-4PACZ/perovskite/PEACl/C_60_/BCP/Ag	1.187	26.10	83.31	25.8					
4-(3,5-Dimethoxyphenyl)butylammonium Chloride (Me-4PACz) (in PVK)	ITO/NiOx/Perovskite/C_60_/BCP/Ag	1.25/1.34	18.3/19.0	84.6/84.2	19.3/21.4	One-step	MPP T80 @ 1,000 h	1.76	Interface induction + Dynamic	Adv. Mater. 2024, 36(41), 2408101
		1.07/1.22	16.5/26.0	59.5/84.6	10.4/26.7(certified26.09)		-	1.56		
ATEMPO (in PVK)	FTO/SnO_2_/perovskite/spiro-MeOTAD/Au	1.178/1.203	25.33/25.68	81.44/81.83	24.30/25.28	One-step	50% RH T90 @2000 h,MPP T92 @ 500 h	1.51	Thermodynamic + Dynamic	Li M. et al. (2025)
Trioctylphosphine oxide (TOPO) (in PbI_2_)	ITO/Self-Assembled Monolayer (SAM)/Perovskite/C60/BCP/Ag	1.22/1.24	21.78/21.76	83.47/84.67	22.18/22.87	Two-step(tandem)	MPP T95 @ 500 h	1.68	Thermodynamic + Dynamic	Nat. Commun., 2025, 16(1), 40
	Si/ITO/2PACz/Perovskite/LiF/C_60_/SnO_2_/IZO/Ag	1.95	19.76	79.95	30.78(certified 30.26)		MPP T95 @ 1,400 h			
4-Fluorobenzylamine hydroiodide (F-PMAI) (in organic salt solution)	ITO/Spiro-TTB/perovskite/C_60_/BCP/Ag	1.10/1.15	22.65/22.77	77.43/85.48	19.38/22.74	Two-step(wide-bandgap)	MPP T90 @ 600 h	>1.65	Thermodynamic + Dynamic	Nano-Micro Lett. 2024, 16(1), 189
	Si/ITO/Spiro-TTB/perovskite/C_60_/SnO_2_/IZO/Ag	1.77/1.81	19.32/20.01	80.11/82.91	27.61/30.05		30%–40% RH MPP T90 @ 700 h			
IPA (as antisolvent)	ITO/SnO_2_/perovskite/Spiro-OMeTAD/Au	1.21/1.21	23.99/23.97	79.9/78.6	23.1/22.8	One-step(wide-bandgap)	MPP T95 @ 1,500 h	1.55–1.78	Interface induction + Dynamic	Liu et al. (2024)
2-amidinopyridine hydroiodide (ADP) (after PVK)	FTO/SnO_2_/perovskite/spiro-MeOTAD/Au	1.180/1.17/1.16	25.39/24.8/25.3	83.7/81.8/83	25.1/23.7/24.5	One-step	20% RH T93 @ 1700 h, MPP T90 @ 1000 h	1.5	Thermodynamic + Dynamic	J. Am. Chem. Soc. 2023, 145(44), 24349–24357
Facet heterojunction	ITO/SnO_2_/(111)perovskite/(001)perovskite/2-Cl-PEAI/spiro-OMeTAD/Au	1.153/1.165	25.26/25.40	81.28/84.23	23.67/24.92	Two-step(evaporate + spin-coat)	MPP T91 @ 2000 h,85 °CT82 @ 1800 h	1.53–1.57	Interface induction	Joule 2025, 9(2),101787

### 3.1 Two-step method

The two-step method, which involves the sequential deposition of a PbI_2_ film followed by conversion via organic ammonium salts, offers a versatile platform for guiding crystal growth through careful manipulation of precursors and intermediates. Its core principle lies in manipulating the crystallography of the PbI_2_ precursor to topotactically steer the orientation of the resulting perovskite layer. The layered structure of the PbI_2_ precursor can be expanded via intercalation additives (e.g., DEACl, BAI), which increase the interlayer spacing. This reduces the nucleation energy barrier of the (111) facet during the subsequent organic ammonium exchange process, thereby promoting its preferential growth.

**FIGURE 2:**
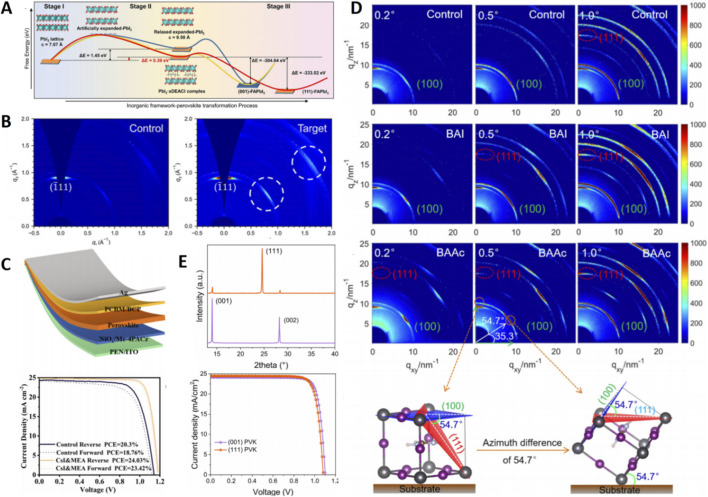
**(A)** Energy evolution of inorganic framework-perovskite transformation process by MD and DFT calculation. **(B)** GIWAXS patterns of control and target perovskite films. **(C)** Schematic layout and J–V curves of flexible PSCs device. **(D)** 2D GIWAXS patterns of the control, BAI- and BAAc-added perovskite films with different incident angles. **(E)** XRD spectra and J-V curves of (111) facet- and (001) facet-dominated PSCs.

An innovation within this framework is the Degradable Additive Complex (DAC) strategy. This approach involves introducing diethylamine hydrochloride (DEACl) and formamide (Fo) into the PbI_2_ precursor (Lin et al., 2025). The insertion of DEA^+^ into the PbI_2_ interlayers expanded the interlayer spacing from 7.08 Å to 9.3 Å, a modification observed via *in situ* XRD to lower the formation energy for (111) α-FAPbI_3_ (−333.02 eV). Crucially, during annealing, Fo and DEACl underwent aminolysis into volatile byproducts that completely escaped, preserving the ideal 1.49 eV bandgap (Figures 2A,B). This process yielded highly (111)-oriented films with grain sizes exceeding 800 nm and significantly mitigated tensile strain due to FA^+^ tilting. Devices fabricated from these films achieved a PCE of 25.2% (0.09 cm^2^) and a certified efficiency of 23.51% (1.04 cm^2^), maintaining 91.68% of their initial performance after 1,080 h of operational stability testing under MPP tracking at 60 °C.

Building on the theme of precursor control, another effective tactic integrates facet engineering within a “PbI_2_ residue control” framework. Co-introducing CsI and melamine (MEA) into the PbI_2_ precursor could achieve this dual goal (Luo et al., 2025). The triazine ring of MEA strongly chelated Pb^2+^, reducing residual PbI_2_, while CsI optimized lattice matching. XRD analysis revealed a sharpening of the (111) diffraction peak (FWHM decreased from 0.18° to 0.12°) and an increase in calculated grain size from 280 nm to 450 nm. TOF-SIMS confirmed MEMA segregation at grain boundaries, effectively passivating Pb^0^ defects (Figure 2C). This yielded single-junction devices with a PCE of 25.66% (certified 25.06%) and flexible devices reaching 24.03%, both retaining over 90% efficiency after 1,000 h of MPP operation.

The mechanistic intricacies of facet control were further unraveled through work elucidating a cation-anion synergistic inhibition mechanism. Research by Liu et al. showed that adding butylammonium iodide (BAI)/butylammonium acetate (BAAc) to the PbI_2_ precursor allowed the larger BA^+^ (4.3 Å vs. FA^+^'s 2.5 Å) to insert into PbI_2_ interlayers, sterically hindering FA^+^ migration along the [001] direction (Liu et al., 2024). Concurrently, Ac^−^ coordination with Pb^2+^ delayed overall crystallization, enabling the (111) orientation to dominate even in the deeper film layers. *In-situ* grazing-incidence X-ray diffraction (GIXRD) showed the (111)/(001) intensity ratio increasing from 1.2 to 2.3 with depth, and 2D-grazing-incidence wide-angle X-ray scattering (GIWAXS) displayed concentrated (111) diffraction spots at χ = 90°, confirming vertical orientation (Figure 2D). These films exhibited reduced defect density, prolonged carrier lifetime (1.2 ns–3.5 ns), and a long diffusion length (L_D_) of 4.23 μm. The corresponding devices achieved a PCE of 25.23% and retained 91% initial efficiency after 1,000 h in 40%–50% RH.

It is important to note that cation composition engineering itself is a potent tool for influencing facet competition. Early work demonstrated that varying the FA/MA ratio altered the (111)/(001) intensity ratio from 0 to 1.7, with (111) gaining relative dominance at lower MA fractions, albeit with some trade-offs in carrier dynamics (Li et al., 2016). To isolate the intrinsic effect of orientation, a subsequent study employed an additive-free two-step method to fabricate both (111)-dominant and (001)-dominant films and devices (Gao et al., 2025). This critical work found that the ultimate photovoltaic parameters (V_OC_, J_SC_, FF, PCE) showed no significant difference, indicating that high performance stems primarily from overall film quality rather than a specific preferential orientation itself (Figure 2E). The defining advantage of the (111) facet lay unequivocally in stability: (111)-films demonstrated superior resilience against thermal degradation (80 °C for 48 h), light-soaking, and high humidity, underscoring that the core benefit of (111) orientation is its exceptional environmental robustness.

### 3.2 One-step method

The one-step spin-coating and anti-solvent method relies on rapid nucleation within milliseconds. Here, additives or green anti-solvents play a decisive role in instantaneously locking in the desired (111) orientation, primarily through additive engineering and anti-solvent engineering. Antisolvent instantaneously extracts DMSO and induces a tilted arrangement of FA^+^ ions, disrupting the thermodynamic stability of the (001) facet and enabling the rapid generate of the (111) facet within milliseconds.

**FIGURE 3:**
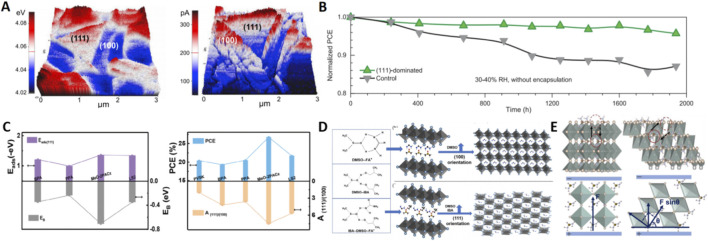
**(A)** KPFM and pc-AFM images of the PPD-driven perovskite film. **(B)** Stability test of the unencapsulated devices for the (111)-dominated perovskite and the control sample stored under 30%–40% RH in air atmosphere for 1938 h. **(C)** Adsorption energy on (111) facet (E_ads (111)_), the dimer binding energy (EB) and photovoltaic efficiency of each studied additive. **(D)** Schematic diagram of the interaction of DMSO with FAI, DMSO with IBA, and DMSO with IBA and FAI, respectively. **(E)** Schematic diagram of the cationic migration route in the (100) and (111) facets.

In the realm of additive engineering, seminal work has demonstrated the power of molecular design. One notable study utilized piperidine (PPD), whose stronger interaction energy with the (111) facet (−0.918 eV) drove its preferential growth (Ma et al., 2022b). Although the work function of the (111) facet (4.07 eV) was slightly higher than (001)’s (4.03 eV), it was lower than (110)’s (4.28 eV), indicating stronger n-type character; more importantly, its photocurrent (165 pA) was sixfold higher than that of the (110) facet (26 pA) (Figure 3A). Devices achieved a quasi-steady-state PCE of 24.64% and retained 96% efficiency after 1,000 h of light-soaking. Further building on this, another research by the same team explored cyclohexylamine (CHA) as additive, forming an intermediate phase with PbI_2_ that facilitated an ultrafast δ→α phase transition within 2 min of annealing, accompanied by a fivefold intensification of the (111) XRD peak (Ma et al., 2023b). Density functional theory (DFT) calculations indicated lower adhesion energy of CHA to the (111) facet, helping to suppress water adsorption (Figure 3B). Films with over 85% (111) coverage yielded devices with 23.8% efficiency, remarkable for retaining 95% initial performance after 1938 h at 30%–40% RH.

A novel kinetic control strategy, termed *in-Situ* Passivation (ISP), offers another avenue for orientation control (Huang et al., 2024). This method uses phosphonic acid molecules that form hydrogen-bonded ion pairs with FAI, preferentially adsorbing onto ammonium-rich (001) facets and inhibiting their growth (Figure 3C). With (001) growth suppressed, the less-adsorbed (111) facet grows preferentially. The extended crystallization time under this method ultimately forms elongated (111)-oriented rod-like microcrystals (>2 μm long), slashing the trap capture cross-section to 8.70 × 10^−26^ cm^2^. This yielded inverted-structure cells with a record 26.7% efficiency (certified 26.09%) and 80% retention after 1,000 h of MPP tracking.

Beyond additives, the choice of anti-solvent itself is a powerful lever. This was convincingly demonstrated by work showing that replacing toxic chlorobenzene with isobutanol (IBA) could effectively induce orientation (Wang et al., 2022). The hydroxyl group of IBA forms asymmetric hydrogen bonds with DMSO, inducing a tilted arrangement of FA^+^ ions that favors (111) orientation (Figure 3D). XRD confirmed this, showing the (111)/(001) intensity ratio jump from 0.8 to 2.1, with vertically percolating grains up to 410 nm. Devices based on three different compositions all surpassed 22% PCE. Complementary research further showed that isopropanol (IPA)/IBA could cleave the edge-sharing PbI_2_·DMSO structure, directly forming corner-sharing (111)-α-FAPbI_3_ (Sun et al., 2023). GIWAXS showed sharp (111) spots, and carrier mobility increased 4.5-fold, leading to zero efficiency decay over 600 h of MPP tracking.

A subtle yet powerful modification involves the introduction of trace water into the anti-solvent process (Qu et al., 2025). DFT calculations revealed that H_2_O molecules adsorb more readily on the (001) facet, hindering its atomic assembly. This selective inhibition allows the (111) facet to grow preferentially (Figure 3E). Beyond growth control, this approach conferred exceptional stability: the migration path for cations in the (111) facet deviates from the electric field direction, resulting in a higher energy barrier (0.22 eV vs. 0.11 eV for (001)) and thus significantly slower ion migration. This translated into highly efficient (26.0% PCE) and ultra-stable (95% retention after 3,500 h) unencapsulated devices that also outperformed under high temperature (85 °C), high humidity (55% RH), and illumination.

### 3.3 Wide-bandgap and tandem cells

The application of (111) facet engineering effectively addresses critical challenges in wide-bandgap (1.65–1.75 eV) perovskites for tandem cells, such as achieving high open-circuit voltage (>1.2 V) and suppressing bromine-induced phase segregation. The high ion migration barrier and superior thermodynamic stability of the (111) facet make it an attractive strategy for enhancing the performance and durability of tandem sub-cells.

**FIGURE 4:**
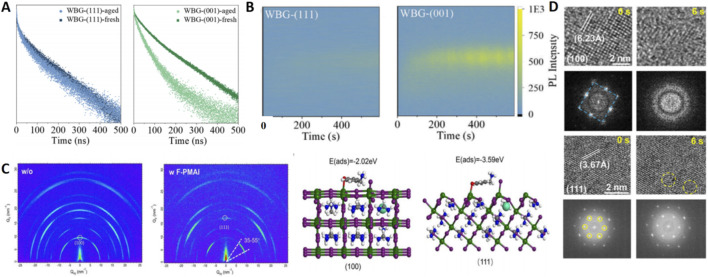
**(A)** TRPL results of WBG perovskite films with (111) and (001) orientation before and after aging. **(B)** *In situ* PL of peak position and intensity of (111) oriented, and (001)-oriented WBG perovskite films with laser irradiation time. **(C)** 2D GIWAXS patterns of the perovskite flms a without and b with F-PMAI. **(D)** Time-sequential HRTEM images of the evolution process of the (100) and (111) plane under continuous electron beam illumination, and the corresponding FFT images.

Li et al. successfully induced (111) dominance in wide-bandgap films using isopropanol (IPA) as an anti-solvent (Li et al., 2024). The (111) facet, rich in Pb dangling bonds, acts as an electron trap, leading to higher electron trap density but consequently higher hole mobility and lower electron mobility compared to the (001) facet. The hydrogen bonding between IPA and FA^+^/I^−^ induces lattice tilting, effectively blocking the straightforward ion migration channels along the electric field direction. *In-situ* PL measurements quantitatively showed that the phase segregation extent at 530 nm under continuous laser illumination was less than half in the (111)-oriented film compared to its (001)-oriented counterpart after 10 min (Figures 4A,B). The wide-bandgap top cell reached 22.8% efficiency and maintained 95% of this after 1,500 h of MPP tracking. Impressively, this strategy demonstrated broad applicability across perovskites with bandgaps ranging from 1.53 eV to 1.77 eV.

Addressing the formidable challenge of depositing high-quality, oriented wide-bandgap films on industrially relevant textured silicon, Liu et al. introduced 4-fluorophenmethylammonium iodide (F-PMAI) into the organic salt solution (Liu et al., 2024). The N-H···F hydrogen bonding from F-PMA^+^ delayed crystallization, while its significantly higher adsorption energy on the (111) facet (−3.59 eV) compared to the (001) facet (−2.02 eV) lowered surface energy and passivated interface defects (Figure 4C). GIWAXS confirmed highly preferential vertical growth of the (111) facet with grains over 1 μm. This resulted in a single-junction top cell efficiency of 22.74% and a four-terminal tandem efficiency of 30.05% (certified 29.4%). Unencapsulated devices retained 90% initial efficiency after 1,650 h in an 85 °C nitrogen environment.

Further demonstrating the versatility of additive approaches, research employing trioctylphosphine oxide (TOPO) as an additive successfully induced preferential (111) growth in 1.68 eV bandgap perovskites (Yao et al., 2025). TOPO’s lower binding energy to the (001) facet selectively inhibited its growth. Striking *in situ* high resolution transmission electron microscope (HRTEM) observations visually confirmed the stability advantage: the (111) facet remained intact under electron beam irradiation, while the (001) facet completely degraded within 6 s (Figure 4D). DFT calculations provided the mechanistic basis, confirming a higher activation energy for I^−^ migration on the (111) facet (1.51 eV vs. 1.13 eV on (001)). The resulting p-i-n tandem cells achieved a remarkable efficiency of 30.78% (certified 30.26%) and exhibited a T_95_ lifetime exceeding 1,000 h under MPP conditions. The team also demonstrated the universality of this method across p-i-n, n-i-p, and semi-transparent device architectures.

Regardless of the one-step or two-step process, the controllable formation of the (111) orientation can be attributed to the coupling of three synergistic mechanisms: Thermodynamic modulation reduces surface energy or expands PbI_2_ interlayer spacing, giving the (111) facet an energetic advantage during nucleation; Kinetic control leverages intermediate-phase stability, delayed crystallization, or instant antisolvent extraction to suppress (001) facet assembly and allow more time for (111) facet growth; Interface induction relies on substrate templating, hydrogen-bonded monolayers, or π-electron passivation to establish chemical gradients at the solid–liquid interface, guiding FA^+^ tilting and Pb–I framework distortion to achieve vertical (111) alignment. However, experimental evidence indicates that most strategies involve multiple mechanisms simultaneously, making it difficult to strictly separate their contributions. Therefore, future work should combine *in situ* thermo-kinetic characterization and machine learning regression to quantitatively decouple the weight of each mechanism. This will establish a predictive theoretical foundation for the rational design of large-area, highly uniform (111)-oriented films.

## 4 Specific optimization strategies for the (111) facet

The control of perovskite (111) facet orientation is currently still in its early stages, transitioning from proof-of-concept to engineering. While the potential of the (111) facet is clear, its practical integration faces the complex reality of polycrystalline films. Achieving pure, homogeneous (111) orientation beyond small-area proof-of-concept devices requires moving past simple promotion of (111) diffraction intensity and towards sophisticated strategies that address inherent heterogeneity and interface defects.

**FIGURE 5:**
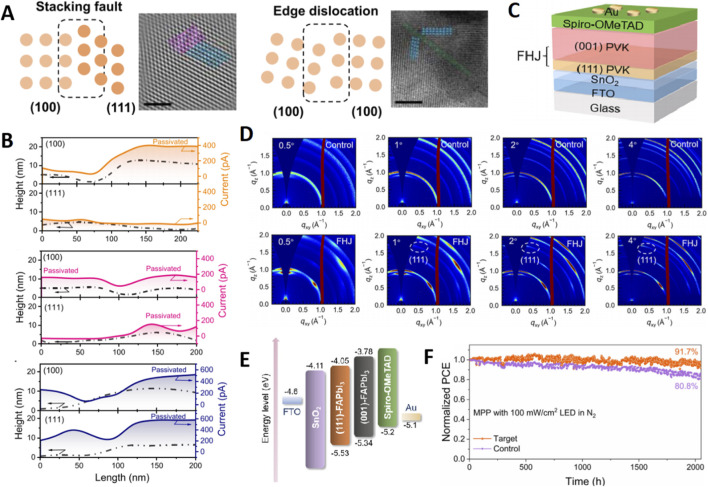
**(A)** Schematics show two typical GB defects that exist in the target film and corresponding TEM images of the GB defects (scale bar: 5 nm). **(B)** Current and height profiles were extracted from the pc-AFM images of the OAI-, PEAI-, and ADP-treated samples, respectively. **(C)** Schematic illustration of the (001)/(111) FHJ. **(D)** 2D GIWAXS patterns of the control (001) and FHJ perovskite films with different incident angles. **(E)** Energy level of each layer in PSCs with FHJ. The energy level alignment references the vacuum level. **(F)** Operational stability of control and FHJ devices up to 2,000 h, respectively (MPP tracking under continuous light irradiation, ISOS-L-1I).

Addressing the unavoidable issue of facet heterogeneity in polycrystalline films has been a key focus. Research has shown that using high-boiling-point, strongly coordinating solvents like NMP in a DMF/DMSO/NMP mixture could lead to preferential nucleation of the (001) facet at the (111) film’s surface and bottom, creating a vertical heterostructure (Zhou et al., 2025). This heterogeneity introduced stacking faults and edge dislocations, degrading performance (Figure 5A). Their solution was a facet homogenization additive, cesium trifluoroacetate (CsTFA), which modulated intermediate-phase competition to transition the film from (111)-dominant to (001)-dominant. Intriguingly, the lowest defect density and optimal device performance were achieved not at pure orientation but when the intensities of the (111) and (001) facets were comparable, highlighting that uniformity of facet distribution can be more critical than extreme purity of a single orientation.

Building upon the understanding that different facets exhibit distinct surface chemistries, a novel “facet-dependent passivation” paradigm has been established (Ma et al., 2023a). They found conventional octylammonium iodide (OAI) was ineffective on the low-polarity (111) facet. In contrast, phenethylammonium iodide (PEAI) utilized the π-electron density of its benzene ring to form strong Pb–π coordination (binding energy −1.859 eV) with Pb^2+^ on the (111) surface, creating an effective bilayer passivation structure (Figure 5B). This insight led to the design of a bifunctional molecule, 2-amidinopyridine hydroiodide (ADP), whose pyridine ring satisfied the π-coordination needs of the (111) facet while its amidinium group addressed the amine-passivation requirements of the (001) facet. This synergistic passivation elevated the current on both facets to ≈600 pA and drastically reduced trap state density.

At the device integration level, a groundbreaking approach was demonstrated through the construction of a (001)/(111) facet heterojunction (FHJ) using a hybrid two-step interdiffusion-evaporation process (Gao et al., 2025). This design cleverly decoupled functionality: a bottom ∼120 nm (111)-oriented layer acted as a stable buffer to suppress ion migration, while an upper ∼600 nm (001)-oriented layer maintained excellent light absorption and charge transport (Figure 5C). GIXRD confirmed a distinct facet gradient through the film depth (Figure 5D). First-principles calculations revealed strong Pb–I bond coupling and a type-II band alignment at the (001)/(111) interface, facilitating carrier separation and suppressing recombination (Figure 5E). The champion FHJ device achieved 24.92% efficiency and demonstrated exceptional stability, retaining 82.6%, 91.7%, and nearly 100% of its initial efficiency under harsh aging conditions of 85 °C, continuous light, and <5% RH storage, far surpassing control devices (Figure 5F). This work exemplifies the potential of strategically leveraging multiple facets within a single device to overcome the limitations of single-orientation films.

## 5 Outlook and discussion

The field of (111) facet orientation engineering, while demonstrating immense promise, is still in a transitional phase from fundamental discovery to scalable integration. Several intertwined challenges must be overcome to fully realize its potential. The primary hurdle remains a insufficient fundamental understanding of the coupled thermodynamic and kinetic factors governing competitive facet growth during the critical early stages of nucleation. This knowledge gap often forces a reliance on empirical screening for new control strategies. Furthermore, successfully translating the exceptional properties of (111)-oriented films from small-area devices to large-area modules is hampered by pronounced center-edge heterogeneity in orientation, driven by rheological gradients and substrate topography during coating processes. Another significant frontier is the extension of these strategies to narrow-bandgap tin-based perovskites, whose facile Sn^2+^ oxidation and narrow crystallization windows demand entirely new, compatible chemical approaches for facet control.

Addressing these challenges will necessitate a multi-faceted toolkit. Technologically, the integration of artificial intelligence for high-throughput virtual screening and the deployment of advanced multi-scale *in situ* characterization techniques will be paramount. Machine learning models can efficiently navigate the vast chemical space of additives, solvents, and annealing protocols to predict optimal conditions for (111) growth. These predictions can be refined using phase-field simulations to visualize nucleation and growth dynamics. Concurrently, establishing a library of facet-specific passivation molecules—featuring modular designs with π-electrons, hydrogen bond donors, or Lewis base groups—will be crucial for targeting the distinct chemical terminations of the (111) facet without compromising orientation quality. A deeper mechanistic understanding will emerge from atomic-scale techniques like *in situ* liquid-phase TEM and GIWAXS, which can unravel the real-time dynamics of facet competition and establish clear structure-activity relationships. At the mesoscale, high spatial resolution surface photovoltage spectroscopy can map and quantify variations in carrier dynamics across different facet regions, ultimately contributing to a unified thermodynamics-kinetics regulatory theory for predictive facet engineering.

Looking beyond current paradigms, future research should explore moving from the pursuit of single-facet dominance towards innovative multi-facet cooperative design. The concept of facet heterojunctions, intentionally creating vertical gradients or lateral patterns of (e.g.) (111) and (001) facets within a single film or device, could harness the high stability of the (111) facet while incorporating the superior charge transport or ideal interface energetics of other low-index facets. This function-by-design approach could break the inherent trade-offs often faced with single orientations. Additionally, external field modulation during processing—using transient temperature gradients, pulsed electric fields, or controlled atmosphere annealing—offers a pathway for dynamic, adaptive reconstruction of facet orientation, potentially optimizing the distribution throughout the film thickness or across the substrate to balance purity with strain management.

For the ultimate goal of industrialization, the key lies in seamlessly integrating (111) orientation strategies with scalable manufacturing techniques like roll-to-roll coating, slot-die deposition, and evaporation-solution hybrid processes. Combining spatial confinement methods with pre-patterned seed crystal arrays could enable the fabrication of quasi-single-crystal films on flexible substrates with minimal grain boundary density. Implementing real-time, *in situ* monitoring systems for stress and optical properties during fabrication could provide the feedback necessary to maintain orientation uniformity across large areas. By tackling these scientific and engineering challenges, (111) facet engineering can evolve from a laboratory curiosity into a cornerstone technology for producing stable, high-efficiency perovskite photovoltaics at the GW commercial scale.
